# Current and Emerging Therapies for Atherosclerotic Cardiovascular Disease Risk Reduction in Hypertriglyceridemia

**DOI:** 10.3390/jcm12041382

**Published:** 2023-02-09

**Authors:** Reed Mszar, Sarah Bart, Alexander Sakers, Daniel Soffer, Dean G. Karalis

**Affiliations:** 1 Department of Physiology, Georgetown University, Washington, DC 20057, USA; 2 Frank H. Netter MD School of Medicine, Quinnipiac University, North Haven, CT 06473, USA; 3 Department of Medicine, Massachusetts General Hospital, Boston, MA 02114, USA; 4 Perelman School of Medicine, University of Pennsylvania, Philadelphia, PA 19104, USA; 5 Department of Cardiology, Thomas Jefferson University Hospital, Philadelphia, PA 19107, USA

**Keywords:** fibrates, lipids, lipoproteins, niacin, omega-3 fatty acids, statins, triglycerides

## Abstract

Hypertriglyceridemia (HTG) is a prevalent medical condition in patients with cardiometabolic risk factors and is associated with an increased risk of atherosclerotic cardiovascular disease (ASCVD), if left undiagnosed and undertreated. Current guidelines identify HTG as a risk-enhancing factor and, as a result, recommend clinical evaluation and lifestyle-based interventions to address potential secondary causes of elevated triglyceride (TG) levels. For individuals with mild to moderate HTG at risk of ASCVD, statin therapy alone or in combination with other lipid-lowering medications known to decrease ASCVD risk are guideline-endorsed. In addition to lifestyle modifications, patients with severe HTG at risk of acute pancreatitis may benefit from fibrates, mixed formulation omega-3 fatty acids, and niacin; however, evidence does not support their use for ASCVD risk reduction in the contemporary statin era. Novel therapeutics including those that target apoC-III and ANGPTL3 have shown to be safe, well-tolerated, and effective for lowering TG levels. Given the growing burden of cardiometabolic disease and risk factors, public health and health policy strategies are urgently needed to enhance access to effective pharmacotherapies, affordable and nutritious food options, and timely health care services.

## 1. Introduction

Normal fasting triglyceride (TG) levels are defined as <150 mg/dL, while mild to moderate hypertriglyceridemia (HTG) is defined as TG levels between 150 to 499 mg/dL and severe HTG as TG levels of ≥500 mg/dL [1]. Mild to moderate HTG is common, with roughly 25% of the U.S. population being affected, while severe HTG occurs in <1% of the population (Table 1) [2,3,4]. Almost all individuals with HTG have a combination of inherited and environmental causes, such as obesity, insulin resistance with or without diabetes mellitus (DM), and fatty liver disease, that collectively contribute to its presence and severity.

Observational and genetic studies have established HTG as an important contributor to atherosclerotic cardiovascular disease (ASCVD) and acute pancreatitis (AP). For this reason, there is a renewed focus on identifying and treating HTG both for primary and secondary prevention. In this review, we discuss the physiology, etiology, and landscape of current and emerging pharmacologic therapies for the treatment of HTG to reduce ASCVD risk.

## 2. Overview of Triglyceride Metabolism

**Figure 1:**
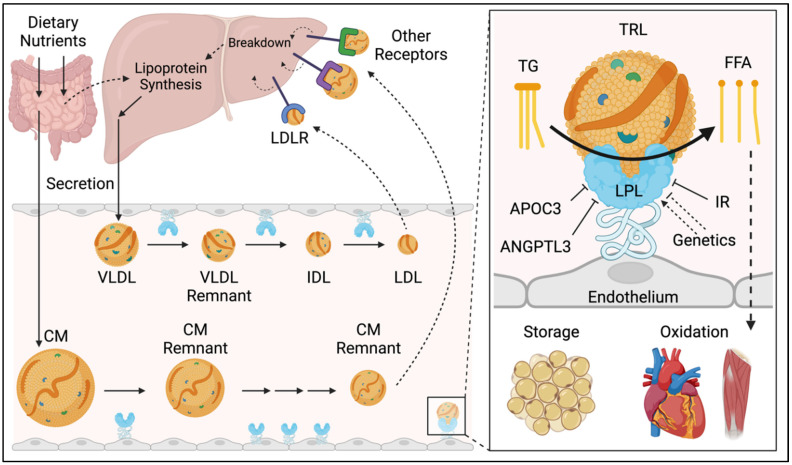
Transport and metabolism of triglyceride-rich lipoproteins. TRL synthesis occurs in both the intestine and the liver. In the intestine, dietary lipids are packaged into chylomicrons (apoB48), and in the liver cholesterol and triglycerides, they are packaged into VLDL (apoB100). Lipoprotein lipase (LPL) releases the TG from TRL by catalyzing the hydrolysis of TG to free fatty acids (FFA). FFA cross the endothelium for oxidation or storage in the underlying tissue. Successive rounds of this process yield denser, triglyceride depleted particles. These particles are taken up by specialized receptors on the surface on hepatocytes, allowing the recycling of their lipid and protein contents. Inset: Several factors inhibit LPL activity, including APOC3 and ANGPTL3, which are novel targets for TG-lowering therapy. Insulin resistance (IR) likewise reduces LPL activity through pleiotropic mechanisms. Genetic variants in LPL or other factors required for its synthesis and functioning also modulate LPL activity. Created with biorender.com (accessed on 26 December 2022). Abbreviations: CM, chylomicron; FFA, free fatty acids; IDL, intermediate-density lipoprotein; LDL, low-density lipoprotein; LDLR, low-density lipoprotein receptor; LDL, lipoprotein lipase; TG, triglyceride; TRL, triglyceride-rich lipoprotein; VLDL, very low-density lipoprotein.

TG has important physiological roles, serving as a fuel source for energy production in skeletal and cardiac muscle and facilitating the storage of excess energy in adipose tissue [5]. TG is trafficked throughout the body in apolipoprotein B (apoB)-containing particles called triglyceride rich lipoproteins (TRL), which carry TG as their principal cargo. TRL have a three-stage lifecycle, consisting of: (1) the production by the liver or intestines, (2) metabolism in the circulation, and (3) clearance by the liver (Figure 1) [6].

In the intestine, dietary lipids are packaged into ApoB48-containing chylomicrons. In the liver, hepatic cholesterol and triglycerides are packaged into ApoB100-containing very low-density lipoproteins (VLDL). Several sources contribute to the hepatic lipid pool, including dietary nutrients (i.e., protein/carbohydrates which are converted to lipids by de novo lipogenesis), circulating free fatty acids, and lipoprotein breakdown.

In the circulation, the enzyme lipoprotein lipase (LPL) releases the TG from TRL by catalyzing the hydrolysis of TG to free fatty acids (FFA). FFA then cross the endothelium for oxidation in skeletal and cardiac muscle or for storage in adipose tissue. Successive rounds of metabolism by LPL yield increasingly dense, more cholesterol rich, and less triglyceride rich particles, termed remnant lipoproteins [7]. Specifically, chylomicrons are metabolized to chylomicron remnants and VLDL are metabolized to VLDL remnants, then intermediate-density lipoproteins (IDL), and eventually low-density lipoproteins (LDL). It is important to note that the term TRL applies to all the aforementioned particles with the exception of LDL. The term “remnant lipoprotein” applies to all partially metabolized TRL.

In the final stage of their lifecycle, particles are taken up by specialized receptors on the surface on hepatocytes, notably the LDL-receptor (LDL-R) and LDL-related receptor 1 (LRP1) among others, which facilitate the recycling of TRL lipid and protein contents. Steady-state TG levels and the abundance of TRL in the circulation are determined by the relative rates of TRL production, metabolism, and clearance. Accordingly, factors which increase production or reduce the clearance of TRL can lead to HTG [8].

## 3. Hypertriglyceridemia and ASCVD Risk

HTG promotes atherosclerosis through several mechanisms. First, HTG reflects elevated TRL concentrations in the circulation and TRL, as with other apoB-containing lipoproteins, are directly atherogenic. In fact, research indicates that nearly all apoB-containing lipoproteins (i.e., those that are small enough to cross the endothelium, with a ~70 nm diameter or less) are approximately equal in atherogenicity on a per particle basis [9]. Second, high plasma TG concentrations promote several characteristic alterations in the circulating lipoprotein profile that are associated with increased atherogenesis. HTG stimulates the activity of cholesteryl ester transfer protein (CETP), which remodels lipoproteins by exchanging TG for cholesterol esters (CE) between TG-rich and TG-poor lipoproteins. This process directly leads to the cholesterol depletion of LDL and high-density lipoprotein (HDL) particles, reducing their particle size and cholesterol content [10]. The resulting small-dense LDL particles are more atherogenic than may be expected from their cholesterol content alone, since there are many molecules of apoB for each unit of cholesterol [9]. Additionally, small cholesterol-depleted HDL particles are more rapidly cleared by the kidneys, further reducing HDL-C and resulting in fewer HDL particles. Overall, in individuals with HTG, although LDL-C cholesterol levels may be normal, non-HDL-C (atherogenic cholesterol) and apoB (number of atherogenic particles) levels tend to be higher, reflecting increased atherogenic risk [11].

Multiple lines of evidence indicate that elevated TRL levels (defined as elevated TG or elevated remnant cholesterol) are associated with ASCVD risk in both primary and secondary prevention, even among statin-treated patients [12,13]. In a recent retrospective study, both primary and secondary prevention participants receiving statin therapy with TG levels of ≥ 150 mg/dL had a lower adjusted risk of death, but a significantly higher risk of major adverse cardiovascular events (MACE) [14]. Fan and colleagues estimated the occurrence of nearly 3.5 million ASCVD events in the next 10 years among individuals with TG levels of ≥ 150 mg/dL [2] and Mendelian randomization studies have provided causal evidence for the role of TG-mediated pathways in coronary heart disease (CHD) incidence [15,16,17].

The atherosclerotic risk associated with TRL is related to the concentration of these atherogenic apoB-containing particles and enhanced by their TG content. Especially in cases of HTG, calculated LDL-C levels may underestimate an individual’s ASCVD risk when there is significant discordance between LDL-C and apoB or non-HDL-C. Consistent with this, non-HDL-C and apoB levels reflect the full risk due to atherogenic lipoproteins better than LDL-C in observational and interventional clinical trials [18]. Therefore, these authors favor using apoB (or non-HDL-C, if not available) levels to estimate the atherogenic risk due in individual patients. However, since national guidelines promote the use of LDL-C as the primary risk assessment and treatment metric, the best methods to calculate LDL-C should be favored. In conventional lipid panels, reported LDL-C levels are calculated from directly measured total cholesterol, HDL-C, and TG levels using the Friedewald formula, which is less accurate when TG levels are high. The Martin–Hopkins table and the Sampson-NIH formula both outperform the traditional Friedewald method and are now preferred up to TG levels of 400 mg/dL [19,20].

Current guidelines for patients with mild to moderate HTG focus on lifestyle modifications and a consideration of statin therapy based on an individual’s cardiovascular risk [21]. For high-risk adult patients with established ASCVD or DM on a maximally tolerated statin whose TG remains elevated, icosapent ethyl omega-3 fatty acids dosed at 4 g a day has been shown to reduce cardiovascular events and should be considered [22].

## 4. Hypertriglyceridemia and Acute Pancreatitis

In addition to the increased ASCVD risk associated with mild to moderately elevated TG levels, HTG is also responsible for up to 15% of AP cases, with the risk and severity of AP increasing in a dose-dependent manner with elevations in TG levels [23,24]. Specifically, the risk of AP in individuals with serum TG levels of >1000 mg/dL is approximately 5%, compared to 10–20% among those with TG levels of >2000 mg/dL [25]. A recent cohort analysis showed that HTG was causative in 7.7% cases of AP, and that TG levels of >11.3 mmol/L (approximately 1000 mg/dL) were associated with a greater incidence of moderately severe AP and longer hospitalization stays [23]. A meta-analysis of 16 studies including nearly 12,000 patients showed that HTG was also associated with pancreatic necrosis and persistent organ/renal failure, and groups of patients with severe HTG had higher rates of complications and mortality for AP [26]. All prior and current guidelines recommend lifestyle modification, along with TG-lowering pharmacologic therapy, for patients with severe HTG to reduce their risk of AP [21]. 

## 5. Genetic and Environmental Causes of Hypertriglyceridemia

**Figure 2:**
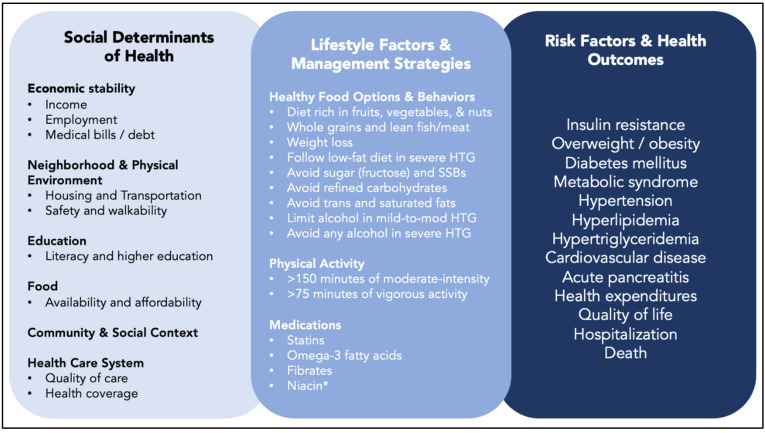
Schematic representation of the proximal and distal factors related to the association between environmental, lifestyle, and social factors contributing to disparities in clinical risk factors and outcomes. Abbreviations: HTG, hypertriglyceridemia; SSB, sugar-sweetened beverages. * Niacin is not recommended for the clinical management of atherosclerotic cardiovascular disease.

Mild to moderate HTG occurs because of inherited and environmental factors. Even in patients with pathologic genetic variants that affect TG metabolism, environmental and modifiable characteristics have an important impact on TG levels and health outcomes (Figure 2). Contemporary dietary patterns contribute directly to HTG, and indirectly by their impact on the development of visceral adiposity, fatty liver, and insulin resistance. Diets composed of calorie-rich, nutrient-poor, fatty, sweetened, and ultra-processed foods contribute to the growing incidence and prevalence of DM, obesity, and HTG.

Monogenic disorders that cause HTG are rare; however, they are more likely to be found in individuals with the most severe HTG (TG levels > 1000 mg/dL) [27]. Recent reports have indicated that severe HTG due to a monogenic disorder occurs with a prevalence of approximately 0.01% in the general population and between 1–2% among all adults with more severe HTG [28,29,30]. Genetic testing is generally not recommended for the identification or treatment of HTG given that the genes regulating TG levels are often recessive with heterogeneity in penetrance [7,31,32]. However, when a monogenic condition such as familial chylomicronemia syndrome, familial lipodystrophy, and familial dysbetalipoproteinemia is suspected, genetic testing may inform disease prognosis, management strategies, and expectations of lifestyle and pharmacologic response.

In addition to genetic and lifestyle factors associated with HTG, secondary causes leading to elevated TG levels may include certain classes of medications including beta blockers, corticosteroids, and antipsychotics, as well as medical and metabolic conditions such as chronic kidney disease, uncontrolled hypothyroidism, and psoriasis (Table 2). Clinicians should assess and address potential secondary causes of HTG when determining appropriate strategies for TG lowering and cardiovascular risk reduction.

## 6. Current Treatments for Hypertriglyceridemia

### 6.1. Lifestyle and Behavioral Modifications

Given the strong association between lifestyle and behavioral factors with elevated TG levels, as well as significant associations between HTG and metabolic syndrome, many of the same treatment approaches for managing insulin resistance, DM, obesity, cardiovascular disease, and fatty liver can be integrated into the care for those with high TG levels. To address the risk of ASCVD associated with HTG, current guidelines recommend an approach to clinical management based on lifestyle strategies, as well as pharmacotherapies utilized for an LDL-C risk-based approach. For patients with severe HTG, lifestyle modifications and pharmacotherapies that specifically lower TG are recommended to reduce the risk of AP. Lifestyle modifications that may result in TG reductions include weight loss (as much as 70% reduction), dietary modifications (including alcohol restriction) (>70%), and physical activity (≤30%) [33,34,35]. Referral to a registered dietitian is strongly recommended to personalize nutrition-based strategies for patients with HTG.

There are different management strategies for optimizing diet, but all should follow healthful approaches that are evidence-based and feasible. Counseling should begin by stressing the elimination or avoidance of caloric-sweetened beverages and ultra-processed foods, along with reviewing what constitutes a heart-healthy diet. Such a diet should be rich in vegetables, fruits, nuts, whole grains, while minimizing simple starches. There should also be a focus on small portions of lean cuts of meat, favoring seafood when possible, and the preference for polyunsaturated (PUFA) or monounsaturated fats as cooking oil in food preparation, as opposed to saturated and trans-fats. Individuals with severe HTG and hyperchylomicronemia (typically with TG levels of >1000 mg/dL), should also reduce total fat from their diet until they have TG levels of <500 mg/dL.

In line with the TG-lowering benefit of PUFA, it is recommended for all individuals to consume ≥2 servings of fish or seafood per week (≥8 ounces), which among other benefits, will increase the intake of omega-3 PUFA and take the place of other less healthy food choices. While fatty fish are recommended for individuals with mild to moderate HTG, those with severe HTG may require leaner seafood options.

It is also important to note the effect of alcohol consumption on TG levels and related metabolic conditions. It has been shown that consuming one ounce of alcohol per day corresponds with a 5–10% higher concentration of TGs when compared with nondrinkers [36]. For individuals with pre-existing HTG, excess alcohol consumption can lead to a substantial increase in TG levels and an increased risk of AP [37]. In patients with severe HTG, it is recommended that they abstain from alcohol use entirely. In addition, a sedentary lifestyle is also associated with HTG, reduced oxidation of muscle fatty acids, and visceral adiposity [21]. Aerobic training has the capacity to decrease TG levels by ~11% and resistance training can lead to reductions of ~6%, though the effect of TG lowering depends on baseline TG levels, caloric expenditure, and intensity/duration of physical activity [38]. It is recommended for adults to engage in ≥150 min per week of moderate-intensity or ≥75 min per week of vigorous-intensity aerobic activity for ASCVD risk reduction [39]. Despite these recommendations, there is unlikely a lower limit on the amount of moderate-to-vigorous physical activity necessary before cardiovascular benefits begin to accrue, so some exercise is always better than none.

### 6.2. Statins

While statins are generally known for their role in decreasing LDL-C levels and reducing individuals’ ASCVD risk, they also provide a 10–30% dose-dependent reduction in TGs in patients with HTG [40]. In patients with severe HTG, with TG levels as high as >800 mg/dL, the dose-dependent effect on the lowering of TG levels with statins has an efficacy of 40–44% [41]. More importantly, clinical trial evidence has shown that individuals with HTG can achieve a meaningful ASCVD risk reduction with statin therapy. The 2018 AHA/ACC/multi-society Guidelines on the Management of Blood Cholesterol consider an elevated TG level of ≥ 175 mg/dL as a risk-enhancing factor and, when present, would favor statin therapy in individuals with a low or borderline 10-year ASCVD risk [1].

However, among statin-treated patients whose LDL-C levels are controlled, elevated TG levels may account for a significant proportion of their residual risk of a recurrent cardiovascular event. In a pooled analysis of 10 clinical trials (N = 5724) of statin-treated individuals with ASCVD, remnant cholesterol was significantly associated with coronary atheroma progression, which was independent of LDL-C, HDL-C, apoB, C-reactive protein, and other clinical risk factors. Higher concentrations of remnant cholesterol, the cholesterol content present in VLDL, is also associated with increased MACE risk [42]. In addition to data derived from clinical trials and meta-analyses, observational cohort studies in statin-treated individuals have also provided key insight into cardiovascular risks correlated with elevated TG levels.

A study using CANHEART (Cardiovascular Health in Ambulatory care Research Team) cohort data from nearly 2.5 million adults in the Ontario population found that nearly one in four statin-treated individuals with ASCVD had HTG and controlled LDL-C levels, and that the risk of ASCVD events increased in a stepwise manner with increasing TG levels [43]. In a post hoc analysis of the TNT (Treating to New Targets) trial, increased TRL levels were associated with a greater cardiovascular risk in patients with stable CHD who had mild to moderate HTG despite their statin therapy. However, more intensive statin therapy (atorvastatin 80 mg) led to greater cardiovascular disease risk reduction in patients with higher TRL levels, which was independent of changes in LDL-C levels [44].

Statins lower LDL-C, but also lower TRL, and higher intensity statins do so more than lower intensity statins. Although statins lower ASCVD risk in patients with HTG, elevated TG levels in statin-treated patients is a marker of residual cardiovascular risk even when the LDL-C is well controlled. Consequently, it is suggested that these individuals may be candidates for interventions such as more intensive lifestyle modifications, high-intensity statins, and high dose icosapent ethyl omega-3 fatty acids, as well as emerging therapies to further reduce their residual cardiovascular risk.

### 6.3. Fibrates

**Figure 3:**
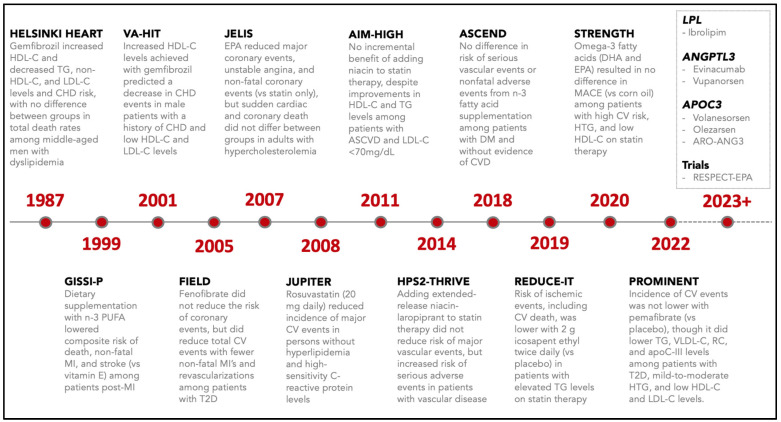
Brief timeline and summary of key clinical trials focused on triglyceride lowering with statins and non-statin therapies (e.g., niacin, fibrates, and omega-3 fatty acids). Abbreviations: ASCVD, atherosclerotic cardiovascular disease; CHD, coronary heart disease; CV, cardiovascular; DHA, docosahexaenoic acid; EPA, eicosapentaenoic acid; HDL-C, high-density lipoprotein cholesterol; LDL-C, low-density lipoprotein cholesterol; MACE, major adverse cardiovascular events; PUFA, poly-unsaturated fatty acids; RC, remnant cholesterol; T2D, type 2 diabetes; TG, triglycerides; VLDL-C, very low-density lipoprotein cholesterol.

Fibric acid derivatives are the most potent TG-lowering pharmacotherapy along with high dose omega 3 fatty acids. They have shown a benefit for ASCVD risk reduction when used as monotherapy, but not when added to statins. Over the past 35 years, there have been a number of key fibrate trials conducted to determine their utility for cardiovascular risk reduction including HHS (Helsinki Heart Study), VA-HIT (VA HDL Intervention Trial), ACCORD (Action to Control Cardiovascular Risk in Diabetes), FIELD (Fenofibrate Intervention and Event Lowering in Diabetes), DAIS (Diabetes Atherosclerosis Intervention Study), BIP (Bezafibrate Infarction Prevention), and most recently the PROMINENT (Pemafibrate to Reduce Cardiovascular Outcomes by Reducing Triglycerides in Patients with Diabetes) trial (Figure 3 and Table 3). A recent systematic review and trial-level meta-regression of nine fibrate trials (N = 41,520), which did not include the results from PROMINENT, concluded that fibrates offer a clinical benefit that is proportional to the degree of non-HDL-C lowering; however, careful consideration should be given to the increased risk of myopathy when added to statin therapy and gemfibrozil should never be combined with statin because of the increased myotoxicity from this combination [45].

Despite null findings from PROMINENT, there are several key takeaways that can inform clinical practice and future research trials. First, given that several post hoc and secondary analyses of previous fibrate trials suggested the clinical benefits of fibrate therapy among individuals with HTG and low HDL-C levels, the subsequent findings that fibrates do not reduce ASCVD events in statin-treated patients with HTG strengthen the case for conducting rigorous studies assessing the validity of post hoc analyses before implementing them into clinical practice [66]. Second, while it confirmed that fibrates should not be used for ASCVD risk reduction in statin-treated individuals, they could still be used to reduce the risk of pancreatitis associated with severe HTG. Third, the PROMINENT trial provided further evidence that, in order for lipid-lowering therapies to show an effect, there needs to be a significant reduction in the levels of apoB-containing lipoproteins [67]. It is suspected that the apoB lowering associated with fibrate therapy is overshadowed by the effect of moderate-to-high intensity statins, thus mitigating the benefit unless used as monotherapy, or unless significant apoB lowering is achieved [68].

### 6.4. Omega-3 Fatty Acids

Omega-3 fatty acids including mixtures of eicosapentaenoic acid (EPA), docosahexaenoic acid (DHA), and purified EPA (icosapent ethyl) have been shown to decrease very high TG levels despite different effects on other physiologic parameters. There have been a number of key studies investigating the role of omega-3 fatty acids for ASCVD risk reduction, including GISSI-P (Gruppo Italiano per lo Studio della Sopravvivenza nell’Infarto miocardico-Prevenzione), JELIS (Japan EPA Lipid Intervention Study), ORIGIN (Outcome Reduction with an Initial Glargine Intervention), ASCEND (A Study of Cardiovascular Events iN Diabetes), REDUCE-IT (Reduction of Cardiovascular Events with Icosapent Ethyl–Intervention Trial), VITAL (the Vitamin D and Omega-3 Trial), STRENGTH (A Long-Term Outcomes Study to Assess Statin Residual Risk Reduction with Epanova in High Cardiovascular Risk Patients with Hypertriglyceridemia), and OMEMI (Omega-3 Fatty Acids in Elderly Patients With Acute Myocardial Infarction). It is important to note that baseline TG levels were not included as part of the eligibility criteria for several of these large trials (Table 3). The recent meta-regression conducted by Marston and colleagues included 13 trials (N = 125,544) on marine-derived omega-3 fatty acids and found that each 1 g/d of EPA administered was associated with a 7% relative risk reduction in major vascular events [45].

The GISSI-P study was conducted in patients with a recent MI (≤3 months) and found that low doses of EPA+DHA was beneficial; however, only a relatively small subset of participants were on statin therapy [54]. Since the GISSI-P study, several large studies including ASCEND, VITAL, and OMEMI have investigated the cardiovascular benefits of low doses of a mixture of EPA+DHA in the statin era, and all failed to show significant reductions in cardiovascular endpoints [58,60,63]. Conversely, JELIS and REDUCE-IT were conducted to examine the effects of moderate to high dose of EPA alone. JELIS was an open-label study of Japanese patients (N = 18,645) with elevated LDL-C on statin therapy, randomized to 2 g of EPA or usual care. Elevated TG levels were not an inclusion criterion in JELIS. Overall, patients randomized to EPA showed a 19% relative risk reduction in major coronary events at a mean follow-up of 4.6 years [55]; however, among participants in the EPA treatment group with HTG and low HDL-C levels, their risk of a major cardiac event fell by 53% [69].

The REDUCE-IT study sought to confirm the findings of the JELIS study and address its limitations in a double-blinded randomized placebo-controlled clinical trial with a higher dose form of purified EPA. The investigators found that icosapent ethyl significantly lowered the risk of ischemic events including cardiovascular death among statin-treated adults (N = 8179) with ASCVD or DM and other risk factors who had mild to moderate HTG and LDL-C levels of 41–100 mg/dL [59]. Analysis of REDUCE-IT found that icosapent ethyl reduced first and then total ischemic events, with well-controlled LDL-C across a range of baseline TG levels, indicating that its observed clinical benefits stem primarily from variation in baseline risk and non-TG-related effects [70].

In contrast to the findings in REDUCE-IT, the STRENGTH trial found that the addition of a carboxylic acid formulation of EPA and DHA (omega-3 CA) resulted in no significant differences in the composite MACE outcome, when compared with the corn oil placebo among statin-treated patients (N = 13,078) with high cardiovascular risk, HTG, and low HDL-C levels [61]. Potential explanations between the differential trial outcomes observed in REDUCE-IT vs. STRENGTH include the different EPA vs. EPA/DHA formulations studied, longer follow-up durations, different placebos utilized (mineral oil vs. corn oil), and different proportions of patients with established ASCVD [21]. To date, icosapent ethyl at high doses is the only omega-3 fatty acid preparation that has been shown to reduce cardiovascular events in high-risk patients with mild to moderate HTG.

## 7. Emerging Treatments for Hypertriglyceridemia

### 7.1. Apolipoprotein C-III Inhibitors

Given the substantial evidence on the association between apoC-III and ASCVD risk, it represents one of the major targets of emerging therapies for TG lowering and cardiovascular risk reduction. Several key genetic and observational studies have shown that *APOC3* loss-of-function mutations are associated with a 40% reduction in TG levels and CHD risk [71,72]. A recent meta-analysis showed that the low risk of ischemic vascular disease observed in *APOC3* loss-of-function heterozygotes is primarily driven by low remnant cholesterol, and not low LDL-C levels, strengthening the case for targeting apoC-III and remnant cholesterol to reduce cardiovascular risk [73]. Additionally, data from a contemporary prospective cohort study (EPIC-Norfolk) found that the top quintile of apoC-III levels predicted CAD risk after adjusting for traditional risk factors and lipid-lowering therapy, but lost statistical significance when adjusted for other lipoproteins. These results suggest that, rather than TG or apoC-III, apoB-containing TRL particles mediate ASCVD risk [74,75].

In 2014, results from the first study to inhibit *APOC3* mRNA (with volanesorsen [previously ISIS 304801]) in humans found that—among three patients with FCS and TG levels between 1406 and 2083 mg/dL—plasma apoC-III levels were reduced by 71% to 90% and TG levels decreased by 56% to 86% [76]. Several years later, in a phase 3, double-blinded RCT of patients with FCS (N = 66), volanesorsen lowered TG levels to <750 mg/dL in 77% of participants; however, a large proportion of patients in the volanesorsen group reported thrombocytopenia and injection-site reactions [77]. To address safety and tolerability concerns, the antisense oligonucleotide (ASO) sequence was combined with a GalNAc moiety to form AKCEA-APOCIII-LRx and inhibit apoC-III protein synthesis in the liver. In a dose-escalation phase 1/2a study in 114 healthy volunteers, AKCEA-APOCIII-LRx (olezarsen) was associated with substantial improvements in the atherogenic lipid profile with only one injection site reaction with erythema, no platelet count reductions, or liver–renal safety signals [78]. Compared to volanesorsen, olezarsen was better tolerated and also associated with favorable changes in lipoprotein concentration and particle size, results that were primarily mediated by decreased TRL levels in patients with mild to moderate HTG who were at high ASCVD risk [79]. It is of note that apoC-III treatment represents the first pharmacologic option to lower TG in FCS patients who are at very high risk for AP, for whom fibrates, omega-3 fatty acids, and statins are usually not effective in lowering TG levels.

### 7.2. ANGPTL3 Inhibitors

In addition to ApoC-III, emerging TG-lowering therapies are also targeting ANGPTL3, given that previous studies have shown loss-of-function variants to be associated with decreased plasma TG and LDL-C levels, as well as a reduction in CHD risk [80,81]. Several pharmacologic compounds that target ANGPTL3 have been developed including the ASO Vupanorsen (previously IONIS-ANGPTL3-LRx) and evinacumab, a monoclonal antibody (mAb) against ANGPTL3.

In terms of the former, recent data from the TRANSLATE-TIMI 70 trial (TaRgeting ANGPTL3 with an aNtiSense oLigonucleotide in AdulTs with dyslipidEmia) showed that Vupanorsen significantly reduced non-HDL-C and TG levels, with modest effects on LDL-C and apoB levels among statin-treated adults (N = 286) with non-HDL-C levels of ≥100 mg/dL and TG levels of ≥150–500 mg/dL [82]. Injection site reactions and elevations in liver enzymes were increased at higher doses of Vupanorsen.

So far, evinacumab investigation has been focused on LDL-C reduction in patients with homozygous familial hypercholesterolemia for whom the FDA has approved its use. Its potential role in the care of individuals with HTG at risk for ASCVD and/or AP is under investigation. However, it has been reported that evinacumab does not lower TG levels in individuals with FCS who lack LPL activity, but may still have an important role in managing moderate and severe HTG in non-FCS patients [83].

## 8. Conclusions

HTG is commonly encountered in the primary and secondary medical care of patients, especially in those with DM and other cardiometabolic conditions, and contributes to a higher risk for ASCVD and AP.

National practice guidelines recognize HTG as a risk-enhancing factor and favor an algorithmic stepwise approach to the care of at-risk patients. All individuals with HTG should be evaluated for and address secondary causes, as well as to undergo individualized lifestyle counseling. Individuals with severe HTG should receive TG-lowering pharmacotherapy, along with lifestyle modification, as primary treatment to reduce AP risk. Those at risk for ASCVD should receive statin-based care, with attention to LDL-C (as well as non-HDL-C and/or apoB), with pharmacotherapy known to reduce ASCVD risk including ezetimibe, proprotein convertase subtilisin-kexin 9 monoclonal antibodies (PCSK9 mAb), and/or a high dose icosapent ethyl esters according to the level of risk and lipid/lipoprotein response. Fibrates, over the counter and prescription mixed formulation omega-3 fatty acids, and niacin have no role in treating elevated TG to reduce ASCVD risk in statin-treated patients in contemporary care, though they may be of value in lowering TG levels in patients with severe HTG to reduce their risk of AP. Patients with HTG often have DM and pharmacologic therapies aimed at treating DM, such as glucagon-like peptide-1 receptor agonists (GLP1-RA), sodium glucose transporter-2 inhibitors (SGLT2i), and metformin, can enable weight loss, improve glycemic control, lower TG, and reduce ASCVD risk.

Novel therapeutics in development including those that target apoC-III and ANGPTL3 appear to be safe, well-tolerated, and effective for the lowering of TG. They may have differing roles in treating individuals with severe HTG and have the potential to offer additional ASCVD risk reduction for those with mild to moderate HTG as well, depending on the results from clinical trials specifically designed to explore this question.

Clinicians must be mindful of the additional burden of pharmacotherapy and the relative powerful impact of lifestyle modifications. The authors acknowledge the important role of personal responsibility for managing lifestyle factors, but stress to our patients and to the readers that we live in a toxic food environment. The current diet in the U.S. has resulted in ~70% of adults with overweight/obesity, ~50% with prediabetes/DM, and ~25% with HTG. Addressing this concern will require a sustained and concerted societal effort beyond what any individual clinician–patient interaction(s) can achieve. However, until that time, clinicians must address care for the patient with HTG with a holistic, empathetic, and individualized approach. In addition to personalized dietary/lifestyle counseling, optimally supported by a registered dietitian nutritionist, appropriate use of evidence-based pharmacotherapies is needed to reduce ASCVD risk.

## Figures and Tables

**Table 1:** Classification of hypertriglyceridemia and risks associated with triglyceride ranges.

HTG Categories	TG Levels (mg/dL)	Lipoproteins	Prevalence *	Clinical Risks	Treatment Approaches
Mild-to-Moderate	150–499	↑ VLDL, TRLs	~1:4–10	ASCVD	Lifestyle/behavioral Statins
Severe	≥500	↑ VLDL, ↑ chylomicrons, or both	~1:10,000	ASCVD + Acute Pancreatitis	Lifestyle/behavioral Very low-fat diet Statins Omega-3 fatty acids Fibrates Niacin

Abbreviations: ASCVD, atherosclerotic cardiovascular disease; HTG, hypertriglyceridemia; TG, triglyceride; TRL, triglyceride-rich lipoproteins; VLDL-C, very low-density lipoprotein cholesterol. * Estimated prevalence based on all individuals with TG levels >150 mg/dL. Note: ↑ indicates increased levels of lipoproteins.

**Table 2:** Genetic disorders and secondary factors (conditions and medications) associated with elevated triglyceride levels and increased hypertriglyceridemia risk.

Genetic Disorders	Secondary Disorders	Medications
Familial combined hyperlipidemia Familial dysbetalipoproteinemia Familial hypertriglyceridemia Multifactorial chylomicronemia syndrome Familial chylomicronemia syndrome Transient infantile hypertriglyceridemia Polygenic hypertriglyceridemia Congenital lipodystrophy	Obesity Metabolic syndrome Diabetes mellitus Hypothyroidism Chronic liver disease Chronic kidney disease Nephrotic syndrome Lipodystrophy Autoimmune disorders Pregnancy (3rd trimester) Weight gain after weight loss Rheumatoid arthritis Glycogen storage diseases Psoriasis Sepsis Multiple myeloma Systemic lupus Cushing syndrome	Beta blockers Thiazides L-asparaginase Bile acid resins Atypical antipsychotics Rosiglitazone Sirolimus Cyclophosphamide Isotretinoin Oral estrogens Tamoxifen Glucocorticoids Retinoids Raloxifene Cyclosporine Interferon Tacrolimus Propofol

**Table 3:** List of key clinical trials investigating non-statin therapies for cardiovascular risk reduction.

Therapeutic Medication Class	Clinical Trials
Fibrates	HHS (1987) [46] BIP (2000) [47] VA-HIT (2001) [48] DAIS (2003) [49] FIELD (2005) [50] ACCORD (2008) [51] LEADER (2016) [52] PROMINENT (2022) [53]
Omega-3 fatty acids	GISSI-P (1999) [54] JELIS (2007) [55] GISSI-HF (2008) [56] ORIGIN (2012) [57] ASCEND (2018) [58] REDUCE-IT (2019) [59] VITAL (2019) [60] STRENGTH (2020) [61] EVAPORATE (2020) [62] OMEMI (2021) [63]
Niacin	AIM HIGH (2011) [64] HPS2-Thrive (2014) [65]

## Data Availability

Not applicable.
